# Chaldean American Views on Plastic and Reconstructive Surgery

**DOI:** 10.1093/asjof/ojag194

**Published:** 2026-10-10

**Authors:** Forrest Bohler, Tamara Attisha, Tyler J Attisha, Tristan Attisha, Elliott Gappy, Sean P Chaiyasate, Varna Taranikanti

## Abstract

**Background:**

Cultural and religious beliefs influence perceptions of plastic surgery, yet attitudes among Chaldean Americans, an ethnoreligious minority from northern Iraq, remain uncharacterized.

**Objectives:**

The aim of this study was to evaluate attitudes toward cosmetic and reconstructive surgery within the Chaldean American community and identify associated cultural, religious, and demographic factors.

**Methods:**

A cross-sectional Qualtrics survey was administered to US Chaldean Americans (age ≥18). Participants were recruited through snowball sampling in metropolitan Detroit. Data on demographics, previous procedures, surgical interest, gender preferences, and religious influence were analyzed using descriptive statistics and sex-based comparisons.

**Results:**

Among 104 respondents (54.8% women, 45.2% men; mean age 27.1), 22.1% reported a previous cosmetic procedure. Women were significantly more likely than men to have had a procedure (31.6% vs 10.6%, *P* = .008) and expressed higher overall interest in future surgery (*P* < .001). Rhinoplasty was the most common procedure among respondents who had undergone cosmetic surgery (65.2%). Men significantly preferred male surgeons for both cosmetic and reconstructive cases (*P* < .001). Religion was cited as a conflicting factor by 35.6% of respondents, with men more likely to report religious influence (*P* = .007). Women demonstrated greater support for all forms of postmastectomy breast reconstruction (*P* = .0017).

**Conclusions:**

In this exploratory survey of Chaldean Americans, attitudes toward plastic surgery varied according to sex and religious beliefs. The prominence of rhinoplasty among respondents parallels trends described in other Middle Eastern populations. Although these findings are not necessarily generalizable to all Chaldean Americans, they provide preliminary insights that may assist surgeons in delivering culturally informed care and support future research in this understudied population.

**Level of Evidence: 3 (Therapeutic):**

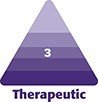

Chaldeans are an ethnoreligious minority group originating from northern Iraq, historically rooted in Mesopotamia and united by their affiliation with the Chaldean Catholic Church, an Eastern Catholic Church in full communion with Rome.^1-4^ For centuries, Chaldeans have maintained distinct linguistic, cultural, and village-based identities, often identifying strongly with their ancestral village, such as Tel Keppe, Alqosh, Ankawa, and Batnaya.^1,3,5^ Although unified by Catholicism, Chaldeans are not a monolithic group. Rather, cultural norms, social expectations, and community structures often vary by original Iraqi village lineage.^6^

Chaldean migration to the United States occurred in several waves during the 20th and early 21st centuries, largely driven by political instability and religious persecution in Iraq. As a result, the United States is now home to one of the largest Chaldean populations in the world, with the large concentrations in the greater Detroit metropolitan region as well as in San Diego County, California.^7,8^ Given that significant migration waves occurred decades ago, there now exist second-, third-, and even fourth-generation Chaldean Americans who were not born or raised in Iraq and whose views may differ from those of first-generation immigrants who directly experienced persecution and displacement.

Despite being a relatively small ethnic minority in the United States with ∼500,000 nationwide, the Chaldean community is socially cohesive and deeply rooted in religious and cultural tradition.^1,2,7,9^ The Chaldean Catholic faith plays a central role in community life, shaping perspectives on modesty, bodily integrity, and medical decision making.^1,2^ In tightly knit communities, family influence, village affiliation, and church networks may exert significant influence over individual healthcare decisions. Given these sociocultural characteristics, it is plausible that beliefs regarding aesthetic and reconstructive surgery may differ from those observed in the broader US population. Yet, to date, the views of Chaldean Americans toward plastic and reconstructive surgery remain largely unstudied.

Previous research has demonstrated that religious and cultural beliefs can significantly influence perceptions of aesthetic and reconstructive surgery in other populations. For example, a questionnaire-based study among Muslim women in Turkey found that nearly one-third of participants believed aesthetic surgery contradicted their religious beliefs, with stronger opposition observed among older individuals and those of lower socioeconomic status.^10^ Similarly, qualitative research among First Nations women in Canada demonstrated that perceptions of breast reconstruction were shaped by cultural identity, spiritual beliefs, and historical experiences, including mistrust of healthcare systems rooted in colonization.^11^ Additionally, qualitative data among American Indian/Alaska Native women revealed culturally specific concerns regarding reconstructive modalities, including opposition to certain materials and a perception of reconstruction as cosmetic rather than restorative.^12,13^

Understanding such perspectives is clinically important. Cultural beliefs influence whether patients seek care, accept reconstructive options, pursue aesthetic procedures, or experience internal conflict regarding surgical decisions. Surgeons who are unaware of culturally rooted concerns may misinterpret hesitation as disinterest, whereas in reality, it may reflect religious or community-based considerations. In ethnically distinct and close-knit communities such as the Chaldeans, where generational status, village identity, and church teachings intersect, these dynamics may be particularly pronounced.

Therefore, we undertook the present survey study to characterize attitudes of Chaldean Americans toward aesthetic and reconstructive surgery. By examining generational status, village heritage, religious beliefs, and perceptions of cosmetic vs reconstructive procedures, this study aims to provide culturally informed insight that may improve patient-centered care within this growing and understudied population.

## METHODS

### Survey Instrument

This cross-sectional survey study was conducted to assess attitudes of Chaldean Americans toward aesthetic and reconstructive surgery. The study protocol was reviewed by the Oakland University IRB and was deemed exempt from formal review because of its anonymous survey design and minimal risk to participants.

The survey instrument was administered electronically using Qualtrics (Provo, UT) from December 2025 to February 2026.^14^ The questionnaire included 15 to 20 questions assessing respondent demographics (age, gender, generational status, village of origin, religious affiliation), as well as attitudes toward reconstructive surgery and elective aesthetic procedures. Both closed-ended (Likert scale and categorical) and open-ended questions were included to allow quantitative analysis and thematic exploration of qualitative responses. Given the exploratory nature of the study and the absence of a previously validated instrument assessing Chaldean American attitudes toward plastic surgery, formal psychometric validation was not performed. However, the questionnaire was reviewed by members of the Chaldean American community before distribution to assess clarity, cultural appropriateness, and content relevance. Feedback from this informal pilot review was used to refine wording and ensure that survey items were understandable and culturally appropriate. The instrument was designed to be exploratory in nature, with the primary aim of establishing baseline attitudes and identifying prevailing themes within the community. The full survey instrument can be found in the Appendix.

### Participant Recruitment

Participants were recruited using a snowball sampling methodology within the Detroit metropolitan Chaldean community. Given the close-knit nature of this population and the absence of a comprehensive sampling frame, this approach was selected to facilitate recruitment of an otherwise difficult-to-access community. Accordingly, the study was designed as an exploratory assessment of attitudes toward plastic and reconstructive surgery, and findings should be interpreted as hypothesis-generating rather than representative of the broader Chaldean American population.

Initial survey distribution occurred through established social, familial, and community networks. Entry into this community was facilitated by multiple members of the authorship team who are themselves members of the Chaldean community, allowing culturally appropriate dissemination and trust-based engagement.

Qualtrics survey links were shared electronically through community contacts and direct messaging. Participants were encouraged to forward the survey to other eligible individuals within their personal networks. Participants were eligible for inclusion if they were 18 years of age or older, self-identified as Chaldean, and resided in the United States.

### Statistical Analysis

Descriptive statistics were calculated for demographic variables and survey responses. Continuous variables were reported as means ± standard deviation (SD) where appropriate, and categorical variables were reported as frequencies and percentages.

Comparative analyses were performed using χ^2^ tests or Fisher's exact tests for categorical variables and *t*-tests for continuous variables as appropriate. A 2-tailed *P*-value of <.05 was considered statistically significant. All statistical analyses and figure generation were performed using Prism GraphPad (Boston, MA).

Qualitative responses from open-ended survey items were analyzed using thematic content analysis. Responses were reviewed independently by study investigators, coded for recurring concepts, and grouped into broader thematic categories (F.B., Ta.A., and Tr.A.). Representative themes were identified to contextualize quantitative findings.

## RESULTS

### Respondent Demographics

A total of 104 respondents completed the survey. The mean age of respondents was 27.1 years (SD 9.5), with a median age of 24 years. The sample consisted of 57 women (54.8%) and 47 men (45.2%). With respect to education, the most common level attained was a bachelor's degree (46.2%), followed by some college (26.9%). Household income varied widely, with the largest proportion reporting annual household income of $150,000 or greater (36.5%). Most respondents reported having health insurance (90.4%). In terms of generational status within the United States, the majority were second-generation Chaldean Americans (59.6%), followed by third-generation (20.2%). Politically, most respondents described themselves as conservative (74.4%; Table 1).

**Table 1.** Respondent Demographics

Demographic	*n* (%)
Sex	
Male	47 (45.2)
Female	57 (54.8)
Annual household income	
Under $25,000	11 (10.6)
$25,000-$49,999	14 (13.5)
$50,000-$74,999	16 (15.4)
$75,000-$99,999	9 (8.7)
$100,000-$149,999	16 (15.4)
Over $150,000	38 (36.5)
Health insurance	
Yes	94 (90.4)
No	10 (9.6)
Previous cosmetic procedure/surgery	
Yes	23 (22.1)
No	81 (77.9)
Highest education level	
Less than high school	4 (3.8)
High school diploma/GED	7 (6.7)
Some college	28 (26.9)
Associates degree	5 (4.8)
Bachelor’s degree	48 (46.2)
Graduate degree	12 (12.0)
Political views	
Very liberal	1 (1.0)
Somewhat liberal	6 (5.8)
Moderate	20 (19.2)
Somewhat conservative	45 (43.3)
Very conservative	32 (30.8)
Age	
Mean	27.1
Median	24
Standard deviation	9.5
Generational status	
First-generation immigrant	17 (16.3)
Second-generation immigrant	62 (59.6)
Third-generation immigrant	21 (20.2)
Fourth-generation immigrant	2 (1.9)
Fifth-generation immigrant (or later)	2 (1.9)

Respondents identified ancestry from several traditional Chaldean villages. The 5 most commonly reported villages were Tel Keppe (66.3%), Alqosh (16.3%), Tesqopa (12.5%), Batnaya (4.8%), and Htara (1.9%). Given the mixed ancestral backgrounds common among Chaldean Americans, respondents were permitted to select multiple ancestral villages. Consequently, percentages for village affiliation exceeded 100%.

### Previous Cosmetic or Reconstructive Surgery

Overall, 27 respondents (26.0%) reported having previously undergone either cosmetic or reconstructive surgery. Of these, 23 respondents (22.1%) reported having a previous cosmetic procedure (Table 1).

Within this sample, cosmetic procedures were substantially more common among women than men. Eighteen women (31.6%) reported having undergone cosmetic procedures compared with 5 men (10.6%). This observed difference between respondent sex was significant (*P* = .008; Table 2).

**Table 2.** Sex-Based Differences in Attitudes Toward Cosmetic and Reconstructive Surgery Among Chaldean American Respondents

Variable	Male (%)	Female (%)	*P*-value
Previous cosmetic procedure	10.6	31.6	.008
No interest in cosmetic procedures	60.0	24.6	<.001
Preference for male cosmetic surgeon	63.2	9.3	<.001
Preference for male reconstructive surgeon	66.0	10.5	<.001
Preference for female cosmetic surgeon	0.0	20.9	.046
Preference for female reconstructive surgeon	4.2	19.3	.018
Support for breast reconstruction after mastectomy	34.0	64.9	.0017
Support for implant-based breast reconstruction	27.7	52.6	.012
Support for autologous breast reconstruction	31.9	57.9	.008
Religious beliefs influence views on cosmetic surgery	44.7	24.6	.007
Cultural traditions influence views on cosmetic surgery	27.7	26.3	.88

Among respondents who had undergone cosmetic procedures, rhinoplasty was the most frequently reported procedure (65.2%), followed by botulinum toxin injections (26.1%) and lip filler injections (26.1%). Rhinoplasty was similarly common among men and women who had undergone cosmetic surgery, reported by 60.0% of men and 66.7% of women.

### Interest in Cosmetic Procedures

When asked about cosmetic procedures they would feel comfortable receiving, 42 respondents (40.4%) reported no interest in any cosmetic procedures. However, interest varied substantially by sex. Sixty percent of male respondents reported no interest, compared with 24.6% of female respondents. A significant difference in reported interest was observed between men and women in this sample (*P* < .001; Figure 1).

**Figure 1.**
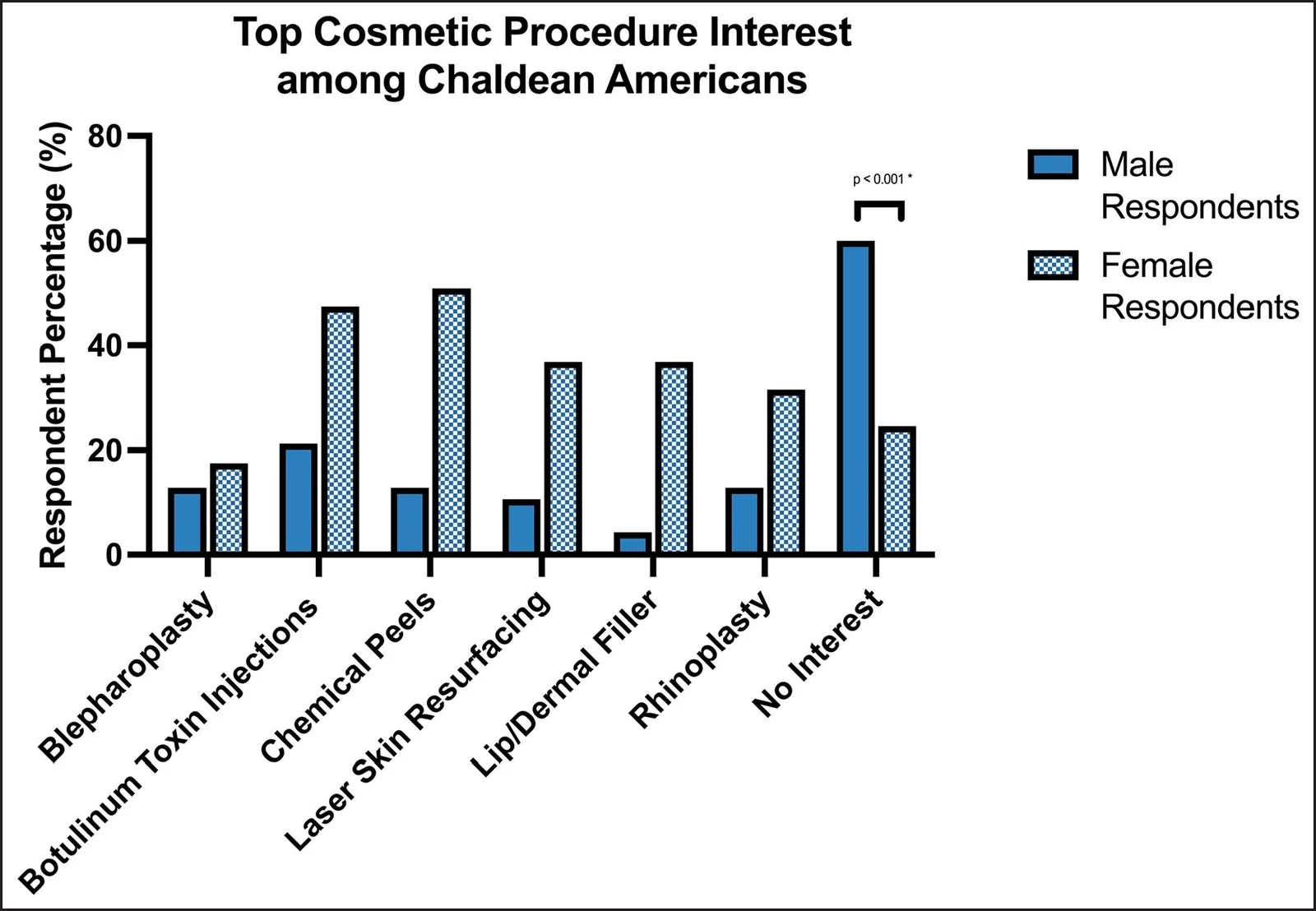
Top cosmetic procedures of interest among Chaldean American respondents by sex.

Among respondents expressing interest in cosmetic procedures (*n* = 62), the top 5 most frequently selected procedures were botulinum toxin injections (59.7%), chemical peels or microneedling (56.5%), and laser skin resurfacing (41.9%), rhinoplasty (38.7%), and lip filler injections (37.1%).

Sex-stratified analysis demonstrated differing procedural interests between men and women. Among men, botulinum toxin injections were the most commonly selected procedure, whereas women more frequently selected chemical peels or microneedling (Figure 1).

### Partner Attitudes Toward Cosmetic Surgery

Among respondents who had undergone cosmetic surgery, 7 respondents reported not having a partner or spouse and were excluded from percentage calculations. Among the remaining respondents (*n* = 16), 50.0% reported that their partner had a neutral attitude, 43.7% reported that their partner had a negative opinion but ultimately left the decision to the participant, and 6.3% reported that their partner had a positive opinion but left the decision to the participant.

### Surgeon Gender Preference

Among respondents expressing interest in cosmetic procedures, preferences regarding surgeon sex varied by respondent sex. Among male respondents (*n* = 19), 63.2% preferred a male surgeon, whereas 36.8% reported no preference and none preferred a female surgeon. In contrast, among female respondents interested in a cosmetic procedure (*n* = 43), the majority (69.8%) reported no preference, whereas 20.9% preferred a female surgeon and 9.3% preferred a male surgeon. Within this sample, men were significantly more likely than women to prefer a male surgeon for cosmetic procedures (*P* < .001; Figure 2).

**Figure 2.**
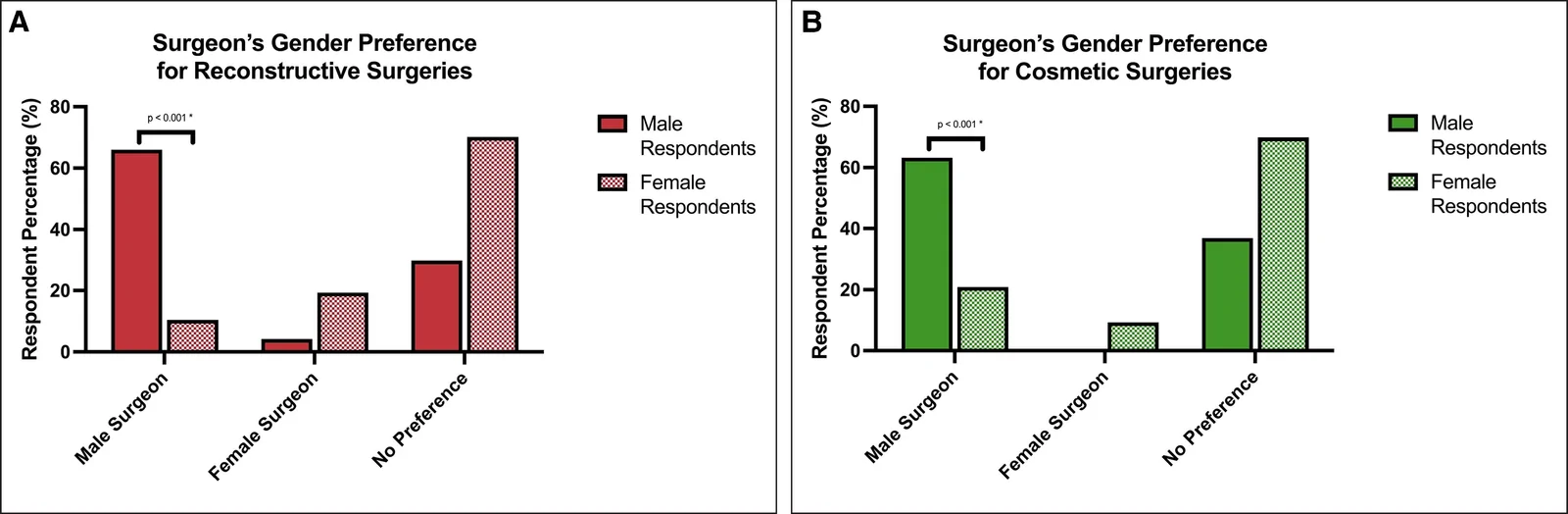
Surgeon gender preference for (A) reconstructive and (B) cosmetic surgeries for Chaldean Americans.

A similar pattern was observed when respondents were asked about surgeon gender preference for reconstructive surgery. Among men (*n* = 47), 66.0% preferred a male surgeon, 29.8% reported no preference, and 4.2% preferred a female surgeon. Among women (*n* = 57), 70.2% reported no preference, whereas 19.3% preferred a female surgeon and 10.5% preferred a male surgeon. Men were also significantly more likely than women to prefer a male surgeon for reconstructive procedures (*P* < .001; Figure 2).

### Religious Considerations

When asked whether cosmetic surgery conflicted with their religious beliefs, 37 respondents (35.6%) reported that it did, whereas 61 (58.7%) reported that it did not. An additional 6 respondents (5.8%) reported that they did not hold religious beliefs. These beliefs were significantly more likely to be cited as an influencing factor by men than by women (*P* = .007).

Qualitative responses from respondents (*n* = 26) who believed cosmetic surgery conflicted with their religious beliefs revealed several recurring themes. The most commonly expressed concern was that cosmetic surgery represents altering the body in a way that conflicts with the belief that humans are created in the image of God. Additional themes included the belief that aging should be accepted as a natural process, concerns that cosmetic procedures encourage vanity or excessive focus on appearance, and the view that reconstructive procedures may be more acceptable than purely aesthetic interventions depending on intent.

### Factors Influencing Attitudes Toward Cosmetic Surgery

Respondents were asked to identify factors influencing their views regarding cosmetic surgery and were allowed to select up to 3 options.

Among male respondents, the most frequently cited influences were personal values or preferences (70.2%), religious beliefs (44.7%), and cultural traditions (27.7%).

Among female respondents, the most commonly cited influences were personal values (64.9%), concern about how their community would perceive them (31.6%), and cultural traditions (26.3%).

### Attitudes Toward Breast Reconstruction

Support for breast reconstruction following mastectomy differed by respondent sex. Among men, 34.0% supported breast reconstruction, 53.2% were neutral, and 12.8% opposed it. Among women, 64.9% supported breast reconstruction, 29.8% were neutral, and 5.3% opposed it. When responses were dichotomized as support vs nonsupport (neutral or oppose), women reported greater support for breast reconstruction following mastectomy than men in this sample (*P* = .0017).

When asked about the cultural or religious acceptability of implant-based reconstruction, 27.7% of men supported the procedure, compared with 52.6% of women, whereas 48.9% of men and 33.3% of women reported neutral views. Women were also significantly more likely than men to support implant-based breast reconstruction (*P* = .012).

Similarly, 31.9% of men and 57.9% of women supported autologous breast reconstruction, whereas approximately half of men (48.9%) reported neutral attitudes. The difference in support for autologous reconstruction was also significant between men and women (*P* = .008).

## DISCUSSION

This study provides initial insight into attitudes toward cosmetic and reconstructive surgery within the Chaldean American community, a population whose perspectives on plastic surgery have not previously been characterized in the literature.

One notable finding was the prominence of rhinoplasty among respondents who had undergone cosmetic surgery. Rhinoplasty accounted for nearly two-thirds of procedures reported for both men and women and remained one of the most frequently selected surgical procedures of interest. This aligns with previous literature describing rhinoplasty as a commonly sought procedure among individuals of Middle Eastern descent because of characteristic nasal features such as dorsal humps, thick skin, and weaker tip support that often motivate patients to seek refinement while preserving ethnic identity.^15-19^ The high prevalence of rhinoplasty observed in this sample may reflect both anatomical considerations and culturally influenced aesthetic preferences.

Although several findings observed in this study are consistent with previous reports from both Middle Eastern and Western populations, the degree to which these findings are unique to Chaldean Americans remains unclear, as the present study did not include a non-Chaldean comparison group.

Several important sex differences were also observed. Women were significantly more likely than men to report previous cosmetic surgery and to express interest in cosmetic procedures. In contrast, men were significantly more likely to prefer a male surgeon for both cosmetic and reconstructive procedures. These findings are consistent with broader cosmetic surgery trends in which women represent the majority of aesthetic surgery patients and do not have a sex preference for their surgeon.^20,21^

Religious beliefs also appeared to play a meaningful role in shaping attitudes toward cosmetic surgery. Over one-third of respondents reported that cosmetic surgery conflicted with their religious beliefs, and men were significantly more likely than women to cite religion as an influencing factor. Qualitative responses frequently framed this concern around the belief that altering physical appearance may conflict with the idea that humans are created in the image of God. However, many respondents distinguished between cosmetic and reconstructive procedures, suggesting that surgeries intended to restore form after disease or injury may be viewed as more ethically acceptable than purely aesthetic interventions.

Attitudes toward breast reconstruction also differed substantially by sex. Women were significantly more likely than men to support reconstruction following mastectomy as well as implant-based and autologous reconstruction. This observed difference may reflect the greater personal relevance of breast reconstruction for women and highlights the importance of culturally sensitive counseling when discussing reconstructive options.

From a clinical perspective, these findings underscore the importance of cultural awareness when caring for patients from Chaldean or other Middle Eastern communities. Surgeons serving these populations may encounter patients whose perspectives on cosmetic surgery are influenced by religious beliefs, cultural norms, or community expectations. In addition, the strong interest in rhinoplasty observed in this study highlights the importance of familiarity with techniques that preserve ethnic nasal identity while addressing aesthetic concerns described in Middle Eastern rhinoplasty literature.^15,16^

### Limitations

This study has several limitations that should be considered when interpreting the findings. First, participants were recruited using a snowball sampling methodology largely within the Detroit metropolitan Chaldean community. Although this approach was necessary to access a relatively close-knit cultural population and facilitated trust-based engagement, it introduces the potential for selection bias, because respondents may share similar social networks, cultural views, or demographic characteristics. As a result, the sample may not fully represent the diversity of perspectives within the broader Chaldean American population given that the majority of respondents identified with Tel Keppe heritage. Furthermore, the study population was relatively young, predominantly second-generation Chaldean American and included a substantial proportion of respondents reporting annual household incomes exceeding $150,000. These demographic characteristics may influence perceptions of cosmetic and reconstructive surgery and may not be representative of the broader Chaldean American population. Consequently, the findings may not fully reflect the views of older individuals, first-generation immigrants, or those from different socioeconomic backgrounds.

Second, because of the nature of snowball sampling and electronic survey distribution through community networks, an accurate response rate could not be calculated. The survey link was distributed through multiple interpersonal channels, and participants were encouraged to forward it to others within their networks, making it impossible to determine the total number of individuals who received the survey invitation.

Finally, although the survey instrument was designed to explore attitudes toward cosmetic and reconstructive surgery within this community, formal survey validation procedures were not performed. The questionnaire was intentionally exploratory in nature and aimed to establish baseline insights into community perspectives rather than to test a previously validated psychometric instrument.

## CONCLUSIONS

This study provides the first characterization of attitudes toward cosmetic and reconstructive surgery within the Chaldean American community. Taken together, these findings suggest that attitudes toward plastic surgery among surveyed Chaldean American respondents may be associated with a complex interaction of cultural, religious, and gender-related factors. These findings provide preliminary insights that may help inform culturally sensitive counseling when caring for patients from ethnically distinct communities and underscore the need for further research examining how cultural identity shapes perceptions of plastic and reconstructive surgery.

## Supplemental Material

This article contains supplemental material located online at https://doi.org/10.1093/asjof/ojag194.

## Supplementary Material

ojag194_Supplementary_Data
