# Supplementary material for: Chaldean American Views on Plastic and Reconstructive Surgery
Source: Aesthet Surg J Open Forum. 2026 Oct 10;8:ojag194. doi: 10.1093/asjof/ojag194 (PMC13696830; doi:10.1093/asjof/ojag194)
Supplement: Supplementary file 1 [file ojag194_supplementary_data.docx]

**Survey Instrument**

**Introduction**

You are being asked to participate in a research study that is being done by Tristan Attisha, fourth-year medical student at Oakland University William Beaumont School of Medicine (OUWB), and Forrest Bohler, fourth-year medical student at OUWB, under the direction of Dr. Varna Taranikanti, Professor in the Department of Foundational Medical Studies at OUWB.

Your decision to participate in this study is voluntary. You can choose to stop your participation at any time or skip any part of the study if you are not comfortable. Your decision will not affect your present or future relationship with Oakland University, the researcher, the Department of Foundational Medical Studies, or Corewell Health.

**What is the purpose of this study?**

This survey is part of a research study exploring views within the Chaldean community about plastic and reconstructive surgery. The goal is to better understand cultural, religious, and personal perspectives related to different types of procedures, including reconstructive care after medical illness and elective aesthetic surgery. Your responses will help inform future patient education, improve cultural sensitivity in surgical care, and guide future research. Participation is voluntary and all responses are completely anonymous.

**Who can participate in this study?**

U.S. citizens who identify as Chaldean and are 18 years of age or older and able to understand English are eligible to participate.

**What do I have to do?**

You will be asked to complete a survey about your views on plastic and reconstructive surgery and how this relates to your cultural values. The survey will be done online and will take approximately 7 minutes to complete. Please respond to the questions as your first honest perception.

**Are there any risks to me?**

For this study, the potential risks are minimal. The survey is anonymous. No identifiable data that can directly be linked to you are collected.

**Are there any benefits to me?**

There may be no direct benefits to you, however, the results of this study may benefit others in the future.

**Will I receive anything for participating?**

You will not receive anything for participating in this study.

**What if I want to stop participating in this study?**

If you want to stop participating, close your browser before clicking ‘submit.’ If you click ‘submit,’ it will not be possible to stop participating.

**Who could see my information?**

Your research records may be shared and reviewed by the following groups:

- Representatives of the Oakland University Institutional Review Board whose job is to protect people who are in research studies and/or other regulatory compliance staff whose job is to facilitate research projects and ensure compliance with the university, state and federal regulations.
- Regulatory authorities who oversee research (Office for Human Research Protections, or other federal, state, or international regulatory agencies)

De-identified data may be used or distributed to another investigator for future research use without additional informed consent from you.

**Who do I contact if I have questions about this study?**

For questions about the study you may contact Dr. Varna Taranikanti (Telephone: 248-370-3886, email: taranikanti@oakland.edu, 444 O’Dowd Hall, 586 Pioneer Drive. Rochester MI 48309). For questions regarding your rights as a participant in human subject research, you may contact the Oakland University Institutional Review Board, 248-370-4898.

-I Agree Checkbox
